# Iterative Design and Usability Testing of the Imhere System for Managing Chronic Conditions and Disability

**DOI:** 10.5195/ijt.2016.6194

**Published:** 2016-07-01

**Authors:** ANDREA D. FAIRMAN, ERIKA T. YIH, DANIEL F. MCCOY, EDMUND F. LOPRESTI, MICHAEL P. MCCUE, BAMBANG PARMANTO, BRAD E. DICIANNO

**Affiliations:** 1 MGH INSTITUTE OF HEALTH PROFESSIONS, SCHOOL OF HEALTH AND REHABILITATION SCIENCES, DEPARTMENT OF OCCUPATIONAL THERAPY, BOSTON, MASSACHUSETTS, USA; 2 UNIVERSITY OF PITTSBURGH SCHOOL OF MEDICINE, PITTSBURGH, PENNSYLVANIA, USA; 3 UNIVERSITY OF PITTSBURGH, DIETRICH SCHOOL OF ARTS AND SCIENCES, DEPARTMENT OF COMMUNICATION, PITTSBURGH, PENNSYLVANIA, USA; 4 UPMC ENTERPISE PROVIDER SOLUTIONS, PITTSBURGH, PENNSYLVANIA, USA; 5 UNIVERSITY OF PITTSBURGH, SCHOOL OF HEALTH AND REHABILITATION SCIENCES, DEPARTMENT OF REHABILITATION SCIENCE AND TECHNOLOGY, PITTSBURGH, PENNSYLVANIA, USA; 6 UNIVERSITY OF PITTSBURGH, SCHOOL OF HEALTH AND REHABILITATION SCIENCES, DEPARTMENT OF HEALTH INFORMATION MANAGEMENT AND BIOMEDICAL INFORMATICS, PITTSBURGH, PENNSYLVANIA, USA; 7 HUMAN ENGINEERING RESEARCH LABORATORIES, VA PITTSBURGH HEALTHCARE SYSTEM, PITTSBURGH, PENNSYLVANIA, USA; 8 DEPARTMENT OF PHYSICAL MEDICINE AND REHABILITATION, UNIVERSITY OF PITTSBURGH SCHOOL OF MEDICINE, PITTSBURGH, PENNSYLVANIA, USA

**Keywords:** Assistive technology, mHealth, Self-management Smartphone, Spina bifida, Telehealth, Telerehabilitation, Usability, Wellness

## Abstract

A novel mobile health platform, Interactive Mobile Health and Rehabilitation (iMHere), is being developed to support wellness and self-management among people with chronic disabilities. The iMHere system currently includes a smartphone app with six modules for use by persons with disabilities and a web portal for use by medical and rehabilitation professionals or other support personnel. Our initial clinical research applying use of this system provides insight into the feasibility of employing iMHere in the development of self-management skills in young adults (ages 18–40 years) with spina bifida (SB) (Dicianno, Fairman, et al., 2015). This article describes the iterative design of the iMHere system including usability testing of both the app modules and clinician portal. Our pilot population of persons with SB fostered the creation of a system appropriate for people with a wide variety of functional abilities and needs. As a result, the system is appropriate for use by persons with various disabilities and chronic conditions, not only SB. In addition, the diversity of professionals and support personnel involved in the care of persons with SB also enabled the design and implementation of the iMHere system to meet the needs of an interdisciplinary team of providers who treat various conditions. The iMHere system has the potential to foster communication and collaboration among members of an interdisciplinary healthcare team, including individuals with chronic conditions and disabilities, for a client-centered approach to support self-management skills.

Mobile health (mHealth) interventions are quickly becoming an alternative means to provide support for self-management for individuals with a variety of chronic conditions such as HIV, as well as cardiovascular and pulmonary diseases (Free et al., 2013). In particular, a significant number of mHealth solutions have been designed to support persons with diabetes (Årsand et al., 2012). While mHealth applications may be successfully implemented with design features appropriate for the general population, there are a large number of persons with disabilities who may require more specialized mHealth solutions. Unique needs such as fine motor difficulties, visual perceptual problems, and sensory and cognitive limitations can greatly impact a user’s ability to utilize mHealth solutions. An example of a specially tailored mHealth solution is the FOCUS system, which was designed to take into account the unique needs of persons with schizophrenia (Ben-Zeev et al., 2013). Creating systems which can support the needs of persons with a variety of disabilities requires a user-centered approach and iterative design process (Rubin & Chisnell, 2008). This article describes the iterative development and testing of the Interactive Mobile Health and Rehabilitation (IMHere) software system.

Smartphones have become widely utilized devices by a variety of persons regardless of gender, age, or disability status. Mobile phone users are typically in the habit of and motivated to carry their phones with them at all times, both at home and in the community. In 2015, the PEW Research Center found that 64% of adults in the United States own a smartphone (Smith, 2015). Smartphones, in particular, facilitate the adoption of new mobile applications. While smartphones are ideal devices due to their pervasive use and portability, their small size creates a variety of challenges in creating interfaces which are easily utilized by persons with disabilities.

Myelomeningocele, the most severe form of SB, is often described as the most complex condition compatible with life (Bowman, McLone, Grant, Tomita & Ito, 2001). Persons born with SB often have a wide range of functional abilities and impairments secondary to their condition which impact daily life, such as loss of motor function, cognitive impairment, visual-perceptual problems, and limited fine motor skills and coordination. Because of the diversity of functional abilities and limitations seen in persons with SB, we determined this population to be an ideal group to involve through surveys (Fairman et al., 2013), usability (Yu, Parmanto, Dicianno, Watzlaf & Seelman, 2015) and feasibility (Dicianno, Fairman, et al., 2015) studies in the creation of the iMHere system. A wide variety of health and self-care needs exhibited by persons with SB may require support (Dicianno et al., 2008). In addition, special consideration and understanding of needs to interface with the iMHere system are necessary to create software applications which may be easily used and incorporated into the daily lives of persons with SB.

The iMHere app initially tested consisted of a suite of six modules providing reminders and monitoring of self-care tasks: (1) MyMeds - medication management, (2) TeleCath – catheterization, (3) BMQs - bowel management, (4) SkinCheck - skin/wound care, (5) Mood - depression screening, and (6) a module designed for nutrition monitoring (Fairman et al., 2013; Parmanto et al., 2013). The consumer is able to record any problems that occur during health maintenance activities (e.g., blood in the urine during catheterization), including reporting on the status of wounds with secure, app-facilitated photographs. The participants’ compliance with health maintenance activities, consumer-reported problems, wound status, and mood survey results are all transmitted to a database which is accessible via a web portal by the consumer’s support person (such as a nurse coordinator or case manager). The support person is able to view this information in real-time or asynchronously and can respond to patients’ needs via the secure portal. The support person is able to respond by contacting the consumer through messaging features within the app, by adjusting the schedule of reminders delivered by the app, or by taking action outside of the system. The combination of interactive, real-time medical monitoring with patient self-care offers a powerful, unique solution to triage the medical problems of patients living with chronic illnesses where cognitive as well as physical disabilities present significant barriers to effective self-care. Data in both directions between the app and the web portal is transmitted and stored using secure, HIPAA-compliant protocols, and data can be erased or locked remotely in the event of device loss. The app modules can work when connected to the portal or in an off-line mode when Internet connectivity is not available (Fairman et al., 2013; Parmanto et al., 2013).

In order for the iMHere system to be an effective intervention, participants will need to effectively utilize the system and adopt the use of this technology as part of their daily routine. Usability is a key factor in adoption of technology as described in Davis’ Technology Acceptance Model (TAM). The TAM describes the adoption and prolonged use of an information system and can be explained by two beliefs: perceived usefulness and perceived ease of use (Davis, 1989). Ultimate success of a telerehabilitation intervention such as this requires that both the consumer app and clinician interface be readily usable.

The primary goal of this work was to evaluate usability of the smartphone app for people with disabilities and usability of the web portal for medical professionals. This usability study was conducted in the context of iterative, participatory development of both the app and the web portal, incorporating lessons learned to refine both components of the system. This paper described three phases of usability testing. Phase 1 focused on initial development of the app, and recruited people with SB to evaluate usability and provide qualitative, formative feedback. This phase of usability testing led into a clinical evaluation of the app described elsewhere (Dicianno, Parmanto, et al., 2015). It was recognized that some persons may have more intensive needs which further limit or prohibit use of their upper extremities in order to operate a commercially available smartphone device. For this reason, more in depth accessibility testing was conducted after the clinical trial was underway and focused on further increasing accessibility of the smartphone application modules (Yu et. al., 2015).

For this clinical study, a researcher used a prototype web interface to interact with the app. The second phase of usability trials, described in the Methods, evaluated the usability of the clinicians’ web portal. Medical professionals were recruited for this phase of testing. Results of these usability trials were combined with formative feedback elicited from consultants representing rehabilitation professionals serving people with SB, spinal cord injury (SCI), traumatic brain injury (TBI), and other conditions. Like the usability trials of the app, usability trials of the web portal were pursued in an iterative manner. Following each round of usability trials, adjustments were made to the web portal user interface. The resulting version of the web portal was then evaluated in the next round of usability trials. This process allowed the team to refine previous features, uncover new barriers, and track the overall progress of the design. Utilizing usability trial results and formative feedback from consultants, adjustments were made to the process of navigating between pages within the web portal; text and error messages were rewritten to be more easily understandable; alerts on a main-page “dashboard” were made more consistent across app modules (e.g., medication, bladder, bowel management, wound care, and mood); additional action buttons were implemented (e.g., to track the progress of a case and provide additional options to send patient messages); role-based access to the portal was implemented; icons were made more consistent across the portal; and features were added to support adding clinical notes and receive SMS text messages for selected portal alerts.

Clinical consultants also provided formative feedback on features of the app which would benefit people with conditions other than SB. Selected features were incorporated in the app. Phase III of usability trials described in this paper evaluated this next version of the app. Participants with a variety of chronic health needs were recruited for this phase.

## METHODS

Usability testing of iMHere 1.0 was completed in three iterative phases:

1. Evaluation of app usability by persons with SB
2. Evaluation of web portal usability for healthcare and rehabilitation professionals
3. Evaluation of the iMHere software system across heterogeneous conditions

Approval was obtained through the University of Pittsburgh’s Institutional Review Board (IRB). All participants signed informed consent prior to the initiation of any recruitment activities for each phase of the usability studies described. Participants were recruited through local health care facilities and community service providers.

## PHASE I: SMARTPHONE APPLICATION USABILITY TESTING IN PERSONS WITH SPINA BIFIDA

**Figure 1:**
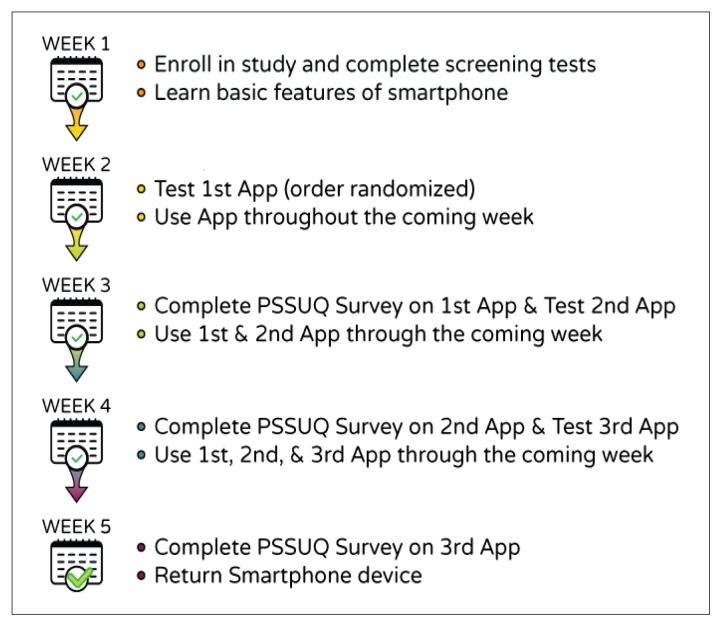
The step-wise process in which usability testing of the iMHere modules occurred over a period of five weeks.

Inclusion criteria were: adults between the ages of 18 and 40 years, a primary diagnosis of SB, history of shunted hydrocephalus as confirmed by a physician, community residence (i.e., not in a group home, personal care home or skilled nursing facility) to help ensure that they would be personally responsible for all or at least some of their self-care tasks. Figure 1 illustrates the step-wise process in which usability testing of the iMHere modules occurred.

Participants completed an adapted version of the MacArthur Competence Tool to ensure that they were able to fully understand the study and able to provide informed consent (Appelbaum & Grisso, 2001). Potential participants needed to score a minimum of eight out of ten to qualify for informed consent. Following informed consent, participants completed several screening tasks. Each participant utilized a Motorola Droid (TM) smartphone device to: (1) place a telephone call, (2) compose and send a specific text message, (3) determine the weather for the following day using a weather app, and (4) report movie show times using the Internet with minimal cues from the researcher. The rationale was that if participants were able to perform all of these tasks successfully, they should also have sufficient fine motor skills, visual perceptual abilities, and cognitive function to be able to successfully utilize the iMHere app modules. Also, during this initial visit, a background survey was completed with each of the usability study participants to gather information related to demographics (age, gender), functional abilities (motor skills and sensory functions), and technical experience. For some of the participants, this was their first experience in using a smartphone.

Participants were instructed in the use of basic smartphone features and were provided the smartphone device to become acclimated during the first week. Usability testing of the app began during the next week, with a new app module being introduced each week. In-person testing was completed in the participants’ homes or other community location of their selection. A researcher provided the participant with instructions on using a given module of the iMHere app, and then provided a series of tasks for the participant to perform. A research assistant monitored the participant’s performance of these tasks on a laptop computer, using Screencast software to capture and record the Android screen as experienced by the participant. Participants were encouraged to “think-aloud” while performing the tasks by verbally sharing their thoughts and opinions. The think-aloud method allowed researchers to determine why the participants were interacting with the software in a particular way and to identify problems with the user interface (Virzi, Sorce & Herber, 1993).

Data for two objective measures were also collected during this usability testing:

- **Completion of Tasks** – User is able to input or retrieve information from the app modules accurately and verbalize when they are “done.”
- **Erroneous Actions** – An action that does not get the user closer to their goal of completing the task.

Task analysis yielded step-wise lists of every single discrete action that a person needed to make in order to utilize the modules. Instances when the person needed assistance to get to the next step were recorded and later reviewed in conjunction with narrative data gathered via audio recordings of the think aloud approach.

**Figure 2:**
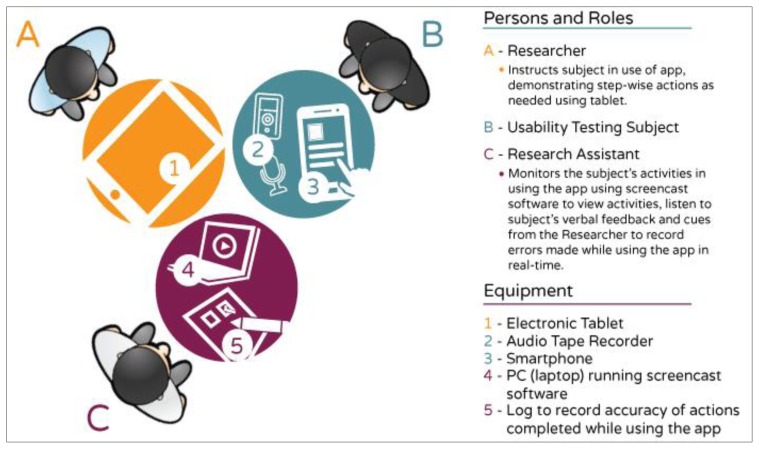
The physical arrangement of the usability testing set-up during Phase I. The research subject was provided a smart phone to utilize the app modules. The researcher provided visual and/or verbal instructions and assistance (if needed) using an electronic tablet. Usability data was gathered via audio recording as well as from a research assistant who was able to monitor the participants’ actions with a laptop screen running screencast software. Importantly, the research assistant was seated across from the participant in order that he or she could observe and document performance without making the participants uneasy or aware of initial errors.

The usability testing set-up is depicted in Figure 2 below:

After testing an app module, each participant utilized it in managing related daily life task(s) over the following week. Each of the app modules was utilized by three participants for at least one week. Subjective self-report measures were then gathered using a survey based on the *IBM Post-Study Usability Questionnaire* (PSSUQ) (Lewis, 1993). The survey used a seven-point Likert scale format with lower numbers correlating with higher levels of satisfaction. Three main categories of subjective data are analyzed through the PSSUQ:

1. **System Usefulness –** Belief that the system will improve the user’s performance.
2. **Interface Quality –** Perception of the user interface layout.
3. **Information Quality –** Belief that a system provides information that is helpful to complete the given tasks.

Open-ended questions were also used to record users’ opinions about the best and worst aspects of the system (Scotch et al., 2007). The app modules were introduced to the participants in a randomized order to help control for practice effects. Usability testing participants trialed a total of three modules during a five-week period. These five-week testing rounds were completed four times in order to conduct usability testing on the six modules previously described.

## RESULTS

A total of seven persons with SB enrolled in this initial phase of usability testing. One person dropped out after the first two weeks of participation due to difficulties with scheduling. The remaining seven participants provided feedback on two iterations of each of the app modules. Table 1 provides a summary of the participants’ characteristics and demographic information.

Results of the modified PSSUQ show an improvement in usability across all of the app modules tested, with the exception of the nutrition module (see Table 2). Limitations in availability of software developers’ time prevented the recommended revisions to the nutrition module. Subsequently, this module was unable to be further refined and was not included as part of the clinical trial of the following phases of usability testing.

## PHASE II: EVALUATION OF WEB PORTAL BY HEALTHCARE AND REHABILITATION PROFESSIONALS

This second phase of usability testing occurred in an effort to commercialize the iMHere system. Investigators representing the company, UbiCue, Inc., met with clinical consultants in a series of structured interviews to compile a list of essential features for the web portal. These interviews were not covered under the IRB and only included health care professionals. These interviews included discussions of how iMHere would fit into clinical workflow and time constraints in real-world settings. Clinical consultants served people with SB, SCI, TBI, multiple sclerosis (MS), cerebral palsy (CP), and other developmental disabilities. They included administrators, neuropsychologists, nurses, occupational therapists, physiatrists, physical therapists, and social workers from the Baltimore and Pittsburgh areas.

The results of these interviews were pooled with feedback from the first round of usability trials and analyzed using qualitative research methods of coding to determine consistent themes. Issues were rated for severity and ease of implementation. This analysis guided refinement of the web portal.

Further feedback was then sought from clinical consultants including administrators, case managers, neuropsychologists, physicians, physical therapists, physiatrists, program coordinators, and speech-language pathologists from the Baltimore, Birmingham, Houston, Memphis, and Pittsburgh areas. Issues for further development were identified, and an initial set of design goals was derived from consultant feedback along with participant feedback and observation of user errors during the initial usability trials. A subset of high priority and practical issues was selected. This list was merged with an existing list of issues identified from prior work, and the final proposed set of design goals was reviewed with consultants.

### WEB PORTAL USABILITY TESTING

#### METHODS

This next component of Phase II was conducted by investigators at the University of Pittsburgh outside of the UbiCue, Inc., corporation to avoid conflict of interest (COI) issues. Approval was obtained through the University of Pittsburgh’s IRB. The web portal was refined in four stages of iterative design separated by five stages of usability testing with clinicians specializing in the care of individuals with complex chronic conditions. At each stage, further redesign was informed by design review with clinical consultants, observations of user behavior during user trials, and participant feedback.

For each round of testing, the web portal was populated with data for fictional clinical scenarios. Participants were provided with a script of routine tasks to perform, requiring them to access data from the portal and react to that simulated data. These tasks included setting up a TeleCath cue, responding to an inbox message, adding a medication reminder, responding to a mood alert, responding to a TeleCath alert, responding to a BMQ alert, and responding to a skin alert. This usability testing required accessing data being entered in real time by an investigator using the iMHere app modules according to a script. Similarly to the Phase I usability testing, participants were encouraged to use the think-aloud method. An investigator observed the participants’ progress and recorded time to complete each assigned task, completion of tasks, and erroneous actions. Subjective self-report measures were gathered using a modified PSSUQ and through open-ended questions. Mann-Whitney U test was used to compare the average PSSUQ scores from Round 1 with those from Round 5, with the null hypothesis being that the average scores would remain equal between the rounds.

To evaluate time to complete tasks, the research team established benchmarks based on clinical expertise for time to complete each of seven tasks which participants were asked to perform during the final round of web portal usability trials. To validate these benchmarks, three of our consultants were also asked to provide benchmarks for the same tasks. All consultants were familiar with the web portal and were rehabilitation professionals with experience with the demands of clinical workflow, particularly for practices serving people with SB, SCI, and TBI. All three consultants’ benchmarks for all seven tasks were equal to or higher than the benchmarks set by the team. Therefore, the benchmarks established by the team were used for analysis.

Open-ended questions recorded users’ opinions about the best and worst aspects of the system, including potential barriers to real-world use in the context of existing clinical workflow. In analyzing the PSSUQ results, to offset responders’ tendency to be polite, a score significantly lower than 2.96 was selected as the target level for each question. For each item of the PSSUQ, 95% confidence intervals were calculated across participants and compared to this target.

#### RESULTS

All participants were employed as clinicians working with individuals with complex and chronic conditions that impair ability to carry out self-management, able to use a computer to access a website, and able to provide informed consent. Twenty-five unique health care and rehabilitation professionals participated in the evaluation of the web portal across five rounds of testing. Twenty-three were female, and two were male; mean age was 38.4 years, standard deviation 12.5 years. The racial and ethnic breakdown was 19 non-Hispanic Caucasian, one Hispanic Caucasian, two African American, and three Asian participants.

### WEB PORTAL: USER RATINGS ACROSS ROUNDS

**Figure 3:**
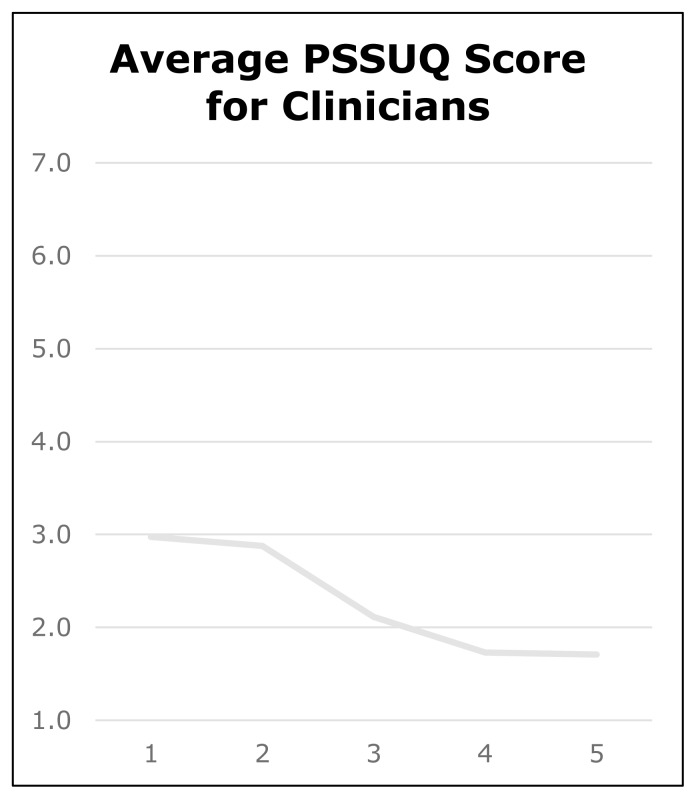
Average PSSUQ scores (average across PSSUQ items for each participant, then averaged across participants) for rehabilitation professionals across five rounds of testing.

PSSUQ ratings consistently decreased favorably over all five rounds from an average total score of 53.0 to 24.9 (total possible range 18–126), with average individual item scores decreasing from 3.0 to 1.7 (see Figure 3). This change in score between round one and round five was significant statistically (p=0.04). By round five, the confidence interval was 0.9 with confidence limits of 0.8 to 2.6.

### WEB PORTAL: FINAL ROUND METRICS

Eleven rehabilitation professionals participated in the final round of user testing: 4 physical therapists, 4 registered nurses, 1 occupational therapist, 1 psychologist, and 1 social worker. All eleven participants successfully completed all seven tasks - set up TeleCath cue, respond to an inbox message, add a medication reminder, respond to a mood alert, respond to TeleCath alert, respond to BMQ alert, and respond to skin alert. Of these, participants made errors in only the ‘respond to BMQ alert’ and ‘respond to skin alert’ tasks, with a maximum of 1 error and mean of .09 errors each.

In the final round of web portal usability testing, for five of seven tasks, the mean time was below the benchmark. For three of seven tasks, the maximum time was below the benchmark. For four of seven tasks, the upper bound of the 95% confidence interval for task completion time was below the benchmark. The worst case was for the maximum time on the response to a mood alert, in which the maximum time was 117 seconds compared to a benchmark of 60 seconds, a difference of 57 seconds.

## PHASE III: EVALUATION OF THE IMHERE SOFTWARE SYSTEM ACROSS HETEROGENEOUS CONDITIONS

### METHODS

The goal of this component of the project was to refine smartphone apps to support people with disabilities other than SB and to incorporate usability results obtained in the previous round of testing. People with chronic disabilities were recruited from local healthcare institutions and community service agencies in the Western Pennsylvania region for usability testing of the patient smartphone app modules to assess whether consumers consider the modules to be easy to learn and use. Each of the participants completed a background survey and screening protocol regarding demographics, functional abilities (motor skills and sensory functions), and technical experience.

Each patient participant took part in a single session. The participant was introduced to the current suite of health maintenance app modules (medication management, catheterization, bowel management, depression screening, skin integrity monitoring) and provided with a list of tasks to perform, requiring them to respond to prompts from the app modules and initiate activities with the app modules. As in Phase I, a think-aloud protocol was again used. The investigator recorded time to complete each assigned task, completion of tasks, and erroneous actions, as described above. Subjective self-report measures were gathered using a modified PSSUQ and open-ended questions. Mann-Whitney U Test was used to compare the means across the two rounds of testing, with the null hypothesis that the means would be equal.

### RESULTS

Thirteen unique consumers participated in the two rounds of evaluation of the app modules. Seven participants were female, six male; mean age was 35.2 years (minimum 18, maximum 64, standard deviation 15.5 years); 10 participants were non-Hispanic Caucasian, one Hispanic, and two African American. Participants’ diagnoses included: Celiac Disease, CP, MS, SCI, SB with/without intellectual disability, SB and Hydrocephalus, and Williams Syndrome.

PSSUQ ratings were low in the two rounds of testing consumers participated in. The average total score ranged from 18.4 to 26.4 (total possible range 13–91), and average individual item scores ranged from 1.4 to 1.8. This change between rounds was not statistically significant. By the last round, the confidence interval was 0.1 with 95% confidence limits of 1.7 to 1.9.

Eight participants took part in the second round of app usability testing (3 with SCI, 3 with CP, 2 with MS). All participants were able to complete all tasks. The maximum number of errors for each participant across all tasks was two errors. Additional details are included in Table 3 below.

## DISCUSSION/SUMMARY

Twenty-three usability issues were identified during usability trials and addressed through refinements to the app modules. The catheterization and bowel management app modules were updated in two ways based on feedback from rehabilitation professionals serving people with SCI. First, to better fit people who perform catheterization and/or bowel management on an as-needed basis rather than on a regular schedule, both app modules were updated to provide users the ability to self-report catheterization or bowel management episodes and any resulting problems. Second, to aid people who are asked to track urine volume or the consistency and size of bowel movements, the ability to report these measures was included into both scheduled and self-reported catheterization and bowel management reports. No refinements to the existing medication reminder, wound tracking, or depression screening app modules were identified specific to any particular diagnosis.

The importance of user-centered design in the development and clinical application of mHealth systems must not be underestimated. This is especially true of systems intended for long-time use to support self-management of chronic conditions. This study utilizes evidence-based methods to assess the usability of the iMHere system with regards to both the web portal on the clinician side and the app modules on the side of persons with disabilities. Feedback from testing participants was incorporated into changes made in subsequent iterations, which exhibited improved usability of this promising new mHealth system.

## Figures and Tables

**Table 1:** Phase I – Participant Demographics

**Enrollment**	N = 7 (1 dropout after 1^st^ round)
**Gender**	4 Males, 3 Females
**Age in years**	Mean = 28.42, SD = 5.32
**Race**	5 - non-Hispanic Caucasians 1 - Hispanic Caucasian 1 - Asian
**Highest Level of Education Completed**	3 - High School Diploma 3 - Some College 1 - Associates Degree
**Received Special Education Services**	2 - no 5 - yes
**Employment Status**	6 - Employed Part-time 2 - Unemployed
**Smart Phone User / Familiar with Apps?**	5 - no 3 - yes

**Table 2:** Comparison of PSSUQ Scores from Phase I Rounds 1 and 2 of Usability Testing

App Modules Tested	Round 1		Round 2	
	Median Scores	Average Range	Median Scores	Average Range
MyMeds	2.0	2.0 – 4.3	1.0	all scored 1.0
TeleCath	1.0	1.0 – 2.3	1.0	all scored 1.0
BMQ’s	1.0	1.0 – 2.7	1.0	all scored 1.0
Mood	2.0	2.3 – 3.0	1.0	1.0 – 3.33
SkinCheck	2.0	1.3 – 3.0	1.0	1.0 – 3.0
NutriCue	1.0	1.3 – 2.0	Not Tested in Round 2	

**Table 3:** Total Errors across All Tasks for Each Participant in the Final Round of App Usability Testing

Diagnosis	Number of Errors
SCI	2
SCI	1
CP	2
CP	0
CP & dyslexia	0
Cervical SCI	0
MS	2
MS	0
